# Tango-Foot

**Published:** 1914-05

**Authors:** 


### "Tango-Foot."

It is doubtful indeed whether the discovery by so eminent a medical specialist as Dr. Gustav F. Boehme, Jr., of New York, of a new disease which he is pleased to term "Tango-Foot," will have the effect of lessening the craze for the newest fancies in the terpsichorean art, but it may be well for physicians to note the diagnosis and the treatment given by Dr. Boehme in the *Medical Record*. He says:

"Occupational diseases have been described for a long time, yet it is only recently that a new affliction, due entirely to our love of the esthetic and the joyous, has laid its hold on the pleasure-loving public. We have known of the bursitis, called 'housemaid's knee'; we have sympathized with the miner with his 'miner's elbow'; the weaver has suffered from that painful condition 'weaver's bottom'—and now our feelings must be extended to the cabaret dancer, the little chorus girl—and to the devotees of Terpsichore in general.

"I have recently been consulted by a number of dancers who came to me complaining of 'pain in the front of the foot,' and in every instance have found the same symptom-complex—and on investigation discovered the cause constant—the modern dance. I feel, therefore, that it will not be amiss to report my experience with this condition.

"*Symptomatology.*—The patient generally awakes in the morning with a slight dull pain in the outer anterior aspect of the leg in its lower third. At first it is regarded as a slight bruise or a 'little rheumatism.' During the next few days the pain becomes more marked and a stiffness in flexion and extension of the foot is noted. Going up and downstairs is painful, especially the latter, He, or she, now becomes worried and seeks medical advice.

"The physician on examination notes the slight limp of the patient. He discovers that the patient finds standing on the toes, or passive and active extension of the ankle joint painful. There is no redness. Over the region of the tibialis anticus tendon pressure produces a slight degree of tenderness. With the hand over this painful area, extension and flexion of the ankle is made and the typical crackling feeling of a tenosynovitis is noticed.

"*Pathogenesis and Pathology.*—The latter day dances, especially the tango and the maxixe, and to some extent the complicated figures of the hesitation waltz call for great flexibility of the ankle, with much movement at this point throughout the various intricate steps. The more common movements are those of extension, flexion, and adduction of the foot. The resultant is a constant strain on the extensor muscles of the foot, viz.: the tibialis anticus, the extensor longus digitorum, and the extensor proprius hallucis, which in turn produces a tenosynovitis in this muscle group. The commonest tendon involved is that of the tibialis anticus.

"*Treatment.*—I have to date seen seven cases of this condition, which I do not report in detail because the symdrome is always the same. One case, of the same clinical picture, I saw in a seamstress who worked the sewing machine with her foot—this is not included in this series—but the condition was the same.

"Rest is the first requisite. By this I mean rest from the exciting cause. Immobilization with plaster of Paris or with adhesive plaster does no good; in fact the pressure irritates, limits walking, and causes needless stiffness. Simple cessation from dancing is all that is necessary, with a limitation in the amount of walking done.

"For the simpler cases massage with alcohol or simple soap liniment is sufficient. If this does not effect the desired result, I find that the following prescription painted over the affected tendon daily will quickly remove the pain:

℞ Tr. Aconiti,

Tr. Belladonnæ, aa, ℥ii.

Tr. Iodi, ℥iv.

Sig.—Paint daily.

"Finally, baking daily for two or three days will remove the effusion in the tendon sheath and restore the limb to its normal.

"I call attention to this condition so that it may not be confused with rheumatism, periostitis, or any other local inflammation. It will pay in every instance when the pain is located as described to ask whether the patient be a disciple of one of the light-footed artists who have added so much joy to the world of late—and apparently have developed a minor pain. If so, look for this tenosynovitis of the tibialis anticus and its allied muscle group which I have called 'Tango-Foot.'"

